# The effect of first step right atrial mapping (FRAM) on ablation duration and fluoroscopy exposure during cavotricuspid isthmus ablation of atrial flutter

**DOI:** 10.3389/fcvm.2023.1205966

**Published:** 2023-06-06

**Authors:** Fu Guan, Ardan M. Saguner, Alexander Breitenstein, Mia Wang, Nadine Molitor, Corinna Brunckhorst, Thomas Wolber, Firat Duru

**Affiliations:** ^1^ Cardiac Arrhythmia and Electrophysiology Division, Department of Cardiology, University Hospital Zurich, Zurich, Switzerland; ^2^ Center for Translational and Experimental Cardiology (CTEC), University of Zurich, Schlieren, Switzerland; ^3^ Department of Biostatistics, University of Michigan, Ann Arbor, MI, United States; ^4^ Center for Integrative Human Physiology, University of Zurich, Zurich, Switzerland

**Keywords:** arrhythmia, atrial flutter, catheter ablation, electroanatomical mapping, outcome

## Abstract

**Aim:**

To investigate the clinical significance of right atrial mapping prior to cavotricuspid isthmus (CTI) ablation in patients with typical atrial flutter (AFL).

**Methods:**

Clinical and ablation parameters were retrospectively assessed and compared in patients undergoing CTI ablation with or without a first-step right atrial mapping (FRAM) by using the CARTO 3D mapping system.

**Results:**

CTI block by radiofrequency ablation (RFA) was achieved in all 143 patients. In the FRAM group there was a shorter ablation duration and fluoroscopy exposure compared with the non-FRAM group. CHA_2_DS_2_-VASc score was associated with higher ablation durations, more ablation applications and increased fluoroscopy exposure. Body mass index (BMI) was associated with longer ablation duration and more ablation applications. Furthermore, patients with reduced left ventricular ejection fraction (LVEF) had longer ablation durations and more fluoroscopy exposure. One patient in the non-FRAM group developed cardiac effusion after ablation. None of the patients had recurrence after 6 months of follow-up.

**Conclusions:**

Patients with high BMI, high CHA_2_DS_2_-VASc score and reduced LVEF may benefit from the FRAM approach by reducing ablation duration, number of ablation applications and fluoroscopy exposure.

## Introduction

Cavotricuspid isthmus (CTI)—dependent atrial flutter (AFL) is common, carries a high thromboembolic risk and can cause tachycardia-mediated cardiomyopathy (1). Radiofrequency ablation (RFA) of the CTI is the first-line treatment recommended by current clinical guidelines (1) with a high success rate. However, as the indications for CTI ablation have become more widespread and the clinical profile of patients more diverse, interventional electrophysiologists may be challenged with difficult CTI ablations, as shown by the wide variability in procedure and fluoroscopy times in such patients in previous publications (2, 3). The current use of three-dimensional (3D) electroanatomical mapping system allows real-time visualization of cardiac anatomy during ablation, providing unprecedented high-quality images, and thus making the real-time visualization of abnormal cardiac structures possible (4).

Activation map of the entire right atrium (RA) by using a 3D electroanatomical mapping system together with entrainment maneuver can prove that the arrhythmia is CTI-dependent. The role of RA mapping performed prior to CTI ablation (first-step right atrial mapping, FRAM) in AFL has not been systematically studied to date. The objective of this study was to investigate whether FRAM might have a positive impact to facilitate CTI ablation procedures in patients with complex anatomies and clinical features.

## Methods

### Study population

This was a single-center, retrospective, observational study that included patients with typical or reverse-typical CTI-dependent AFL who underwent an RFA procedure at the University Heart Center Zurich between January 2018 and December 2022. The procedures were all performed by experienced electrophysiologists in our team. We reviewed the procedural characteristics and outcomes of two different groups: the group of patients who underwent RA electroanatomical mapping prior to CTI ablation (first-step right atrial mapping, FRAM group) and the group of patients who underwent direct CTI ablation without prior RA electroanatomical mapping (non-first-step right atrial mapping, non-FRAM group). All enrolled patients had ongoing AFL at the time of the RFA procedure. We did not include patients who underwent pulmonary vein isolation during the same procedure. Patients who were unavailable for clinical follow-up within six months after the procedure were not included.

This study complied with the Declaration of Helsinki regarding investigation in humans. All patients gave written consent for data use.

### Ablation procedure and follow-up

All RFA procedures were performed under local anesthesia. Analgesic sedation was used, if deemed clinically necessary. A steerable decapolar electrode catheter was used to record coronary sinus (CS) signals during AFL. A 3D electroanatomical mapping system (CARTO, Biosense Webster) was used for mapping and ablation in all patients. If necessary, fluoroscopic imaging was performed using a biplane fluoroscopy system (BICOR HS, Siemens Medical Systems). The system was uniformly set to pulse mode at 3 to 7.5 frames per second with a diameter of 23 cm image intensifier. Collimation and magnification were not utilized during the procedure.

In the FRAM group, electroanatomical mapping of RA during AFL was performed prior to CTI ablation to confirm a CTI-dependent AFL and identify the RA anatomy. A high-density mapping catheter (Pentaray, Biosense Webster) was used in this group. In the non-FRAM group, CTI-dependent atrial flutter was diagnosed on the basis of the clinical electrocardiogram in combination with entrainment maneuver. The local anatomy of the CTI in non-FRAM group was obtained retrospectively by reviewing point-by-point ablation line along the CTI by ablation catheter. The presence of a pouch in this study was defined as a depression (>2 mm in depth) within the CTI line in both groups. The pouch depth was measured both at right anterior oblique (RAO) and left anterior oblique (LAO) projections.

After confirmation of CTI-dependence, point-by-point ablation was performed from the tricuspid annulus (TA) to the inferior vena cava (IVC). An ablation catheter (Thermocool SmartTouch, Biosense Webster) was used in all patients. Mean contact force of 10 to 15 grams was targeted during ablation in both groups. During RF application, a uniform VISITAG module was used to mark the lesions. The settings were as follows: stabilization range of 25%, contact force (CF) of 3 g, label diameter of 3 mm, and point distance of 6 mm. The ablation power was set to 35–40 W. Saline flushing was set to 30 ml/min. Ablation lesion assessment was evaluated with target ablation index of 600 in combination with local potential amplitude reduction more than 50%, or elimination of local signals before ablation index reached 600. In the two groups, CTI ablation was performed using the same ablation catheter and with the same power and VISITAG settings.

All procedures were performed during AFL. After termination of AFL, RFA was continued during pacing from the proximal CS to achieve bidirectional CTI block. In the absence of first-round conduction block, the location of the conduction gap was determined by achieving bidirectional conduction block with additional RF application. Gaps were annotated to mark the location on the CARTO system. During a 20 minute waiting time, CTI block was repeatedly checked during CS proximal pacing. In the presence of reconnection, the gap was again marked and additional RF applications were applied.

After the procedure, all patients had bed rest for 6 h, and 12-lead ECG was recorded before discharge. A clinical follow-up was scheduled after 6 months with surface and Holter ECGs and for evaluation of symptoms.

### Statistical analysis

Categorical variables were analyzed using the chi-square statistics and expressed as percentages. Quantitative variables were present as means with standard deviation, as appropriate for their distribution. Wilcoxon test was used for continuous variables, Chi-square for categorical variables, and Fisher's exact test for small cells. All statistical analyses were performed using the SPSS statistics (version 20.0, IBM Corp., Armonk, NY, USA). Correlation of various measurements were assessed using Spearman's correlation coefficient (R). A *P* value <0.05 was considered statistically significant for all tests.

## Results

### Patients characteristics

The baseline characteristics of the patients are summarized in Table 1. The median age was 65 ± 12 years, with CHA_2_DS_2_-VASc score was 2.1 ± 1.4. There were no differences in the baseline characteristics between the two groups.

**Table 1:** Baseline patient characteristics.

	All (143)	non-FRAM (79)	FRAM group (64)	*P* value
Age (years)	65 ± 12	65 ± 12	66 ± 12	0.73
Male sex (*n*)	116	49	67	0.52
BMI (kg/m^2^)	27.1 ± 4.5	27.6 ± 4.7	21.3 ± 2.2	0.20
BSA (ft2)	21.4 ± 1.9	21.5 ± 1.7	21.3 ± 2.2	0.50
Hypertension (*n*)	91	51	40	0.80
Coronary artery disease (*n*)	44	28	16	0.18
Other cardiomyopathies (*n*)	11	7	4	0.56
COPD (*n*)	43	26	17	0.41
History of AFib (*n*)	26	14	12	0.87
History of PVI (*n*)	5	2	3	0.66
AFL cycle length (ms)	250.1 ± 25.9	249.6 ± 15.6	250.8 ± 34.7	0.28
Other AFL during ablation (*n*)	11	7	4	0.14
Left atrial enlargement (*n*)	110	55	55	0.03
Right atrial enlargement (*n*)	61	36	25	0.50
CHA_2_DS_2_-VASc (IQR)	2.1 ± 1.4	1.9 ± 1.4	2.3 ± 1.4	0.10
Creatinin (umol/l)	101.5 ± 43.9	100.9 ± 48.1	102 ± 38.5	0.49
NT-proBNP (pg/mL)	2,591 ± 1,093	2,506 ± 985	2,706 ± 1,227	0.08
LVEF (%)	50.7 ± 11.1	50.1 ± 11.3	51.3 ± 11.0	0.26
**Medication (*n*)**				
Amiodarone	12	7	5	0.22
Beta-blocker	45	30	15	0.15
Ic anti-arrhythmic drugs	3	2	1	0.60
Oral anti-coagulation	98	60	38	0.37
Anti-platelet agent	35	24	11	0.44

NT-proBNP, brain natriuretic peptide; CHA_2_DS_2_-VASc [congestive heart failure, hypertension, age (≥65 = 1 point, ≥75 = 2 points), diabetes, previous stroke/transient ischemic attack (2 points)]; LVEF, left ventricular ejection fraction rate.

### Procedural outcomes

#### 3D-mapping characterizations

**Figure 1:**
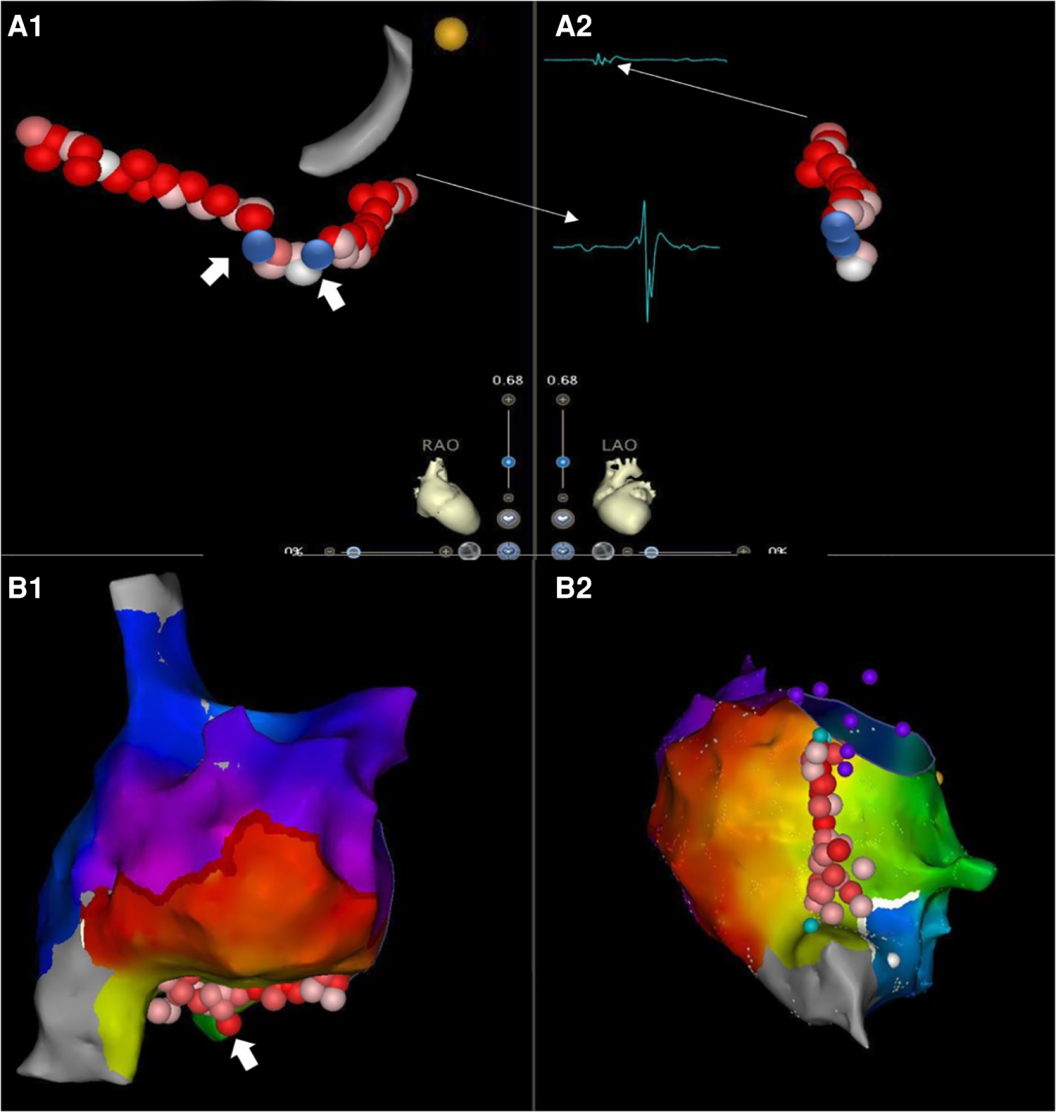
CTI ablation guided by 3D mapping system with minimized fluoroscopic exposure. Cavotricuspid isthmus ablation with continuous lesions in AFL patients with/without prior RA high-density mapping [(**A**). non-FRAM vs. (**B**). FRAM)] (RAO, right anterior oblique view; LAO, left anterior oblique view). The starting point for CTI length was defined as 7 o'clock direction on LAO perspective, with the first local activation signal of annulus on TV side on RAO view (small **A** and big V, A1 narrow arrow). The end point for CTI length measurement on TV side was defined as 6-7 o'clock direction on LAO perspective with the last local activation signal of atrium (small **A**) on the Cava side (RAO) (A2, narrow arrow). The CTI was firstly identified a pouch by high-density map in FRAM group, as shown in B1 (white thick arrow). The CTI was later identified a pouch by ablation line in a patient from non-FRAM group, as shown in A1 (white thick arrow). The contiguous lesions are shown in a light red color on the screen, the blue dot was marked as the gap detected due to failure of first-pass block, or a reconnection during the waiting time after ablation, which was shown located in the pouch area in (**A**).

The CTI length was measured as the distance between the TA and IVC in RAO view (Figure 1). The median length of CTI was 29.1 mm ± 5.9 mm. The presence of a pouch was revealed by CARTO mapping before ablation in the FRAM group or by ablation catheter mapping during CTI ablation in non-FRAM group.

#### Ablation parameters

**Figure 2:**
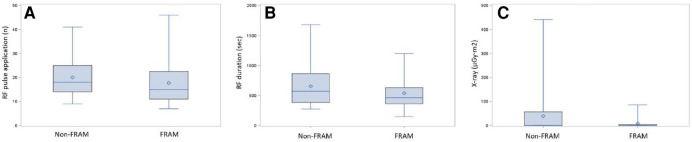
Comparison histogram of radiofrequency parameters in two groups. The distribution of ablation parameters in both groups including RF pulse applications (**A**), RF duration (**B**) and fluoroscopic exposure (**C**) Data were shown in Table 2.

The mean RA mapping time was 12.5 ± 3.8 min in the FRAM group. There was no difference in the total procedural time between two groups, which was recorded from the start of 3D mapping to the end of the CTI ablation. Fourteen patients (21.9%) revealed at least one pouch by CARTO mapping before ablation in the FRAM group and sixteen patients (23.0%) revealed pouches by mapping during CTI ablation in the non-FRAM group (Table 2). Median ablation duration was 602 ± 303 s. Ablation duration in the FRAM group was significantly shorter compared with in the non-FRAM group (*P *< 0.05). In addition, patients in this group also received less RF applications (*P *< 0.05). The fluoroscopy exposure during ablation was lower in the FRAM group than in the non-FRAM group (*P* < 0.05, Figure 2).

**Table 2:** Comparison of 3D mapping and ablation radiofrequency data.

	All (143)	Non-FRAM (79)	FRAM (64)	*P* value
**3D mapping data**				
CTI length (mm)	29.1 ± 5.9	29.6 ± 6.0	28.6 ± 5.8	0.29
RA mapping time (min)			12.5 ± 3.8	
Total procedural time (min)	66.6 ± 21.0	65.9 ± 20.5	67.5 ± 21.7	0.60
Pouch (*n*)	30 (21.0%)	16 (20.3%)	14 (21.9%)	0.81
**Radiofrequency ablation data**				
RF pulse applications (*n*)	19.0 ± 8.2	20.0 ± 8.1	17.7 ± 8.1	0.04
RFA duration (sec)	601.5 ± 302.9	653.2 ± 327.4	537.8 ± 258.2	0.04
Fluoroscopic exposure (µGy.m^2^)	58 ± 24.9	73.7 ± 38.5	17.8 ± 8.1	0.005
Fluoroscopic duration		0.4 ± 0.7	0.08 ± 0.03	0.005

**Figure 3:**
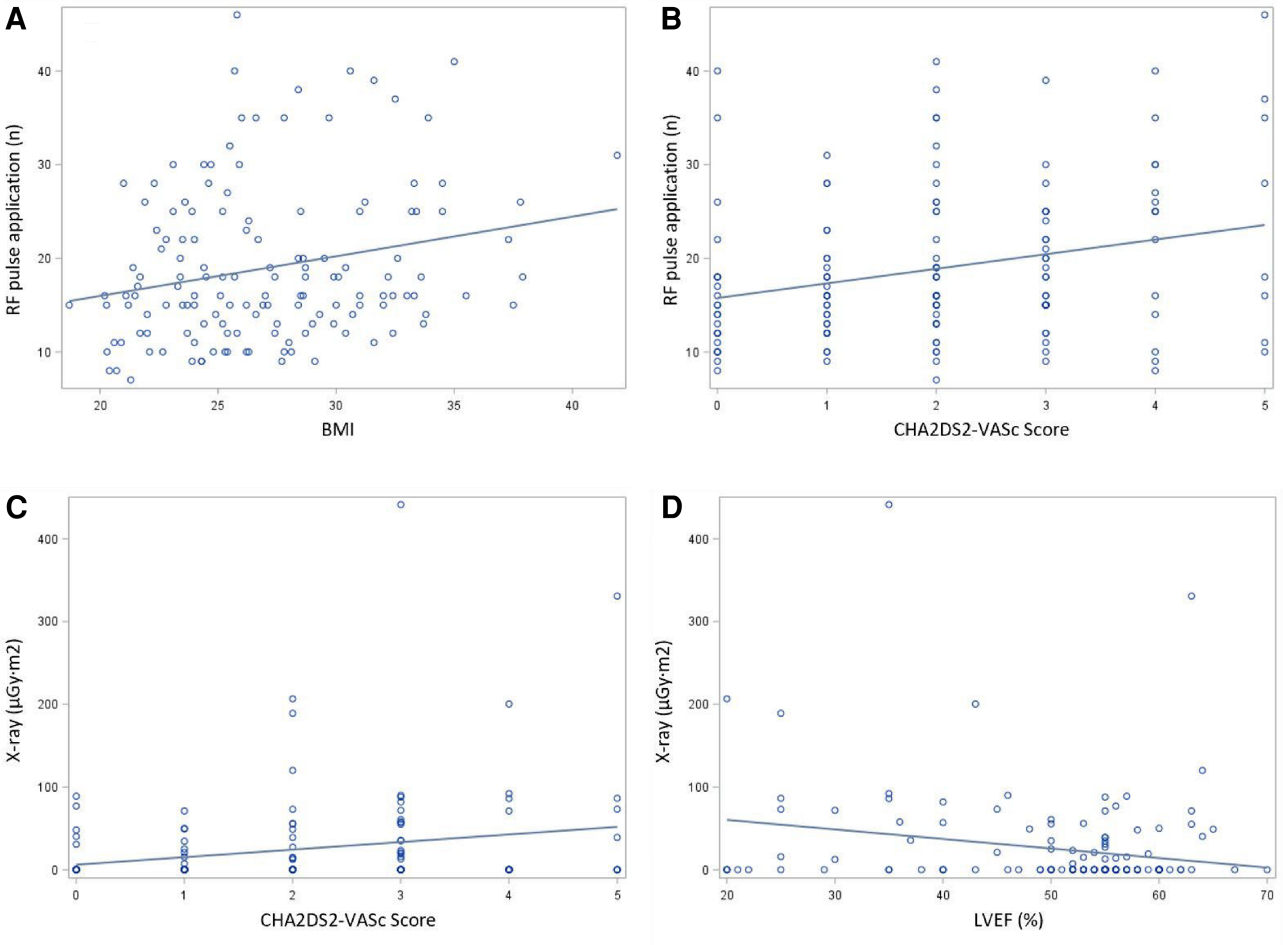
Correlation presentation by spearman coefficient (**A**) Association between patients’ clinical features and ablation parameters determined by coefficient with regression line. (**A**) The relationship between BMI and number of RF application. (**B**) The relationship between CHA_2_DS_2_-VASc score and number of RF application. (**C**) The relationship between CHA_2_DS_2_-VASc score and fluoroscopy exposure. (**D**) The relationship LVEF and fluoroscopy exposure.

**Figure 4:**
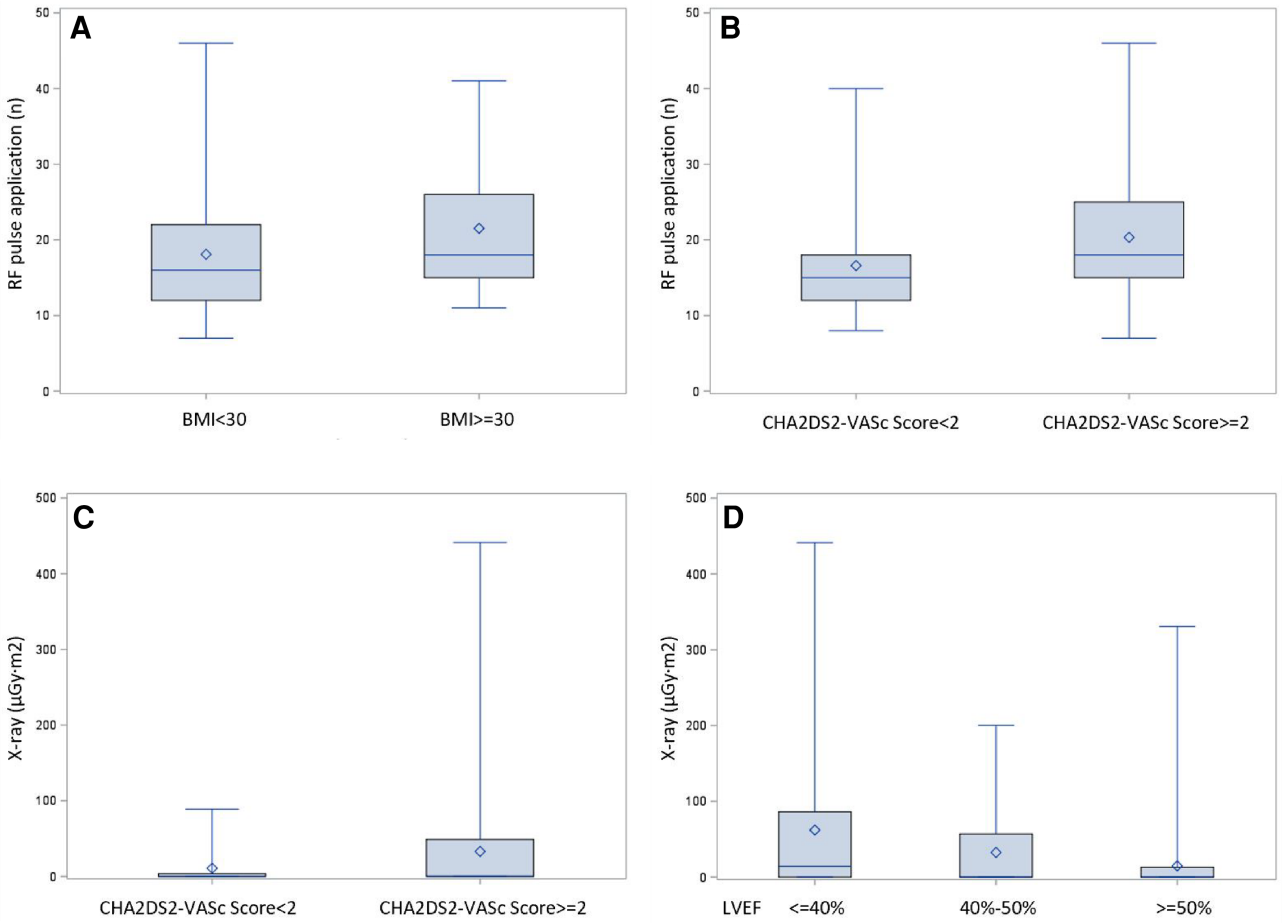
Comparison presentation of dichotomous correlation factors. Comparative analysis between patients with different clinical features. (**A**) Obese patients with BMI ≥30 received more RF applications than with normal body weight (21.5 ± 8.5 and 18.1 ± 7.9, *P* = 0.0094). (**B**) Patients of CHA2DS2-VASc score ≥ 2 received more RF applications compared with patients of CHA2DS2-VASc score<2 (20.3 ± 8.7 and 16.6 ± 6.7, *P* = 0.0054). (**C**): Patients of CHA2DS2-VASc score ≥ 2 had more fluoroscopic exposure compared with patients of CHA2DS2-VASc score<2 (69.5 ± 33.0 µGy.m^2^ and 22.2 ± 10.7 µGy.m^2^, *P* = 0.0206). (**D**): Patients with normal, mild reduced or severely reduced LVEF manifested a trend of increased fluoroscopic exposure (40.0 ± 14.7 µGy.m^2^, 48.0 ± 32.6 µGy.m^2^, 104.0 ± 62. 2 µGy.m^2^, respectively, *P* = 0.0004).

Univariate and multivariate linear regression analysis were performed to identify the independent correlates of ablation duration, ablation pulse application and fluoroscopy exposure. CHA_2_DS_2_-VASc score was associated with higher ablation durations (*r* = 0.21, *P* < 0.05), more ablation applications (*r* = 0.24, *P* < 0.05) and increased fluoroscopy exposure (*r* = 0.20, *P* < 0.05). Body mass index (BMI) was associated with longer ablation durations (*r* = 0.80, *P* < 0.05) and more ablation applications (*r* = 0.22, *P* < 0.05). Furthermore, patients with reduced left ventricular ejection fraction (LVEF) had longer ablation durations (*r* = 0.55, *P* < 0.05) and more fluoroscopy exposure (*r* = 0.19, *P* < 0.05) (Figure 3). Meanwhile, we performed a comparative analysis between patients who were overweight and those with a normal body weight, and patients with higher CHA_2_DS_2_-VASc score and lower CHA_2_DS_2_-VASc score, as well as a trend test between patients with severe LVEF reduction, mild LVEF reduction and normal LVEF. Figure 4 showed the results, which were consistent with the above correlation analysis.

Reconnection during the waiting time occurred in 6 of 64 (9%) patients in the FRAM group and in 9 of 79 (11%) patients in the non-FRAM group (*P* = 0.55). Reconnected gaps were located at the pouch area in 11 of 15 (73%) patients (Figure 1B). Right AFL aside from CTI-dependent AFL was induced during electrophysiological study after CTI ablation in 4 of 64 (6%) patients in the FRAM group and in 7 of 79 (9%) patients in the non-FRAM group. Among those patients with another right-sided AFL, 5 of 7 (71%) were remapped with the mapping catheter.

None of the patients developed cardiac tamponade during ablation, and only one patient in non-FRAM group developed asymptomatic medium cardiac effusion after ablation and recovered with conservative management. Bidirectional block was achieved in all patients at the end of the procedure. None of the patients had clinical or ECG recurrence of AFL after 6 months of follow-up in both groups.

## Discussion

Our study demonstrated that in patients with CTI-dependent AFL, real-time guided linear ablation with RA mapping (FRAM) prior to RFA was associated with reduced ablation durations and applications as well as fluoroscopy exposure during ablation. For patients with high BMI, high CHA_2_DS_2_-VASc scores and reduced LVEF, these benefits were more prominent.

A number of studies have recently reported CTI anatomical abnormalities, such as pouches detected by 3D transesophageal echocardiography (TEE), intracardiac ultrasound (ICE) and cardiac magnetic resonance imaging, which were associated with prolonged ablation times and energy delivery during CTI ablation (5–7). The authors pointed out that anatomical abnormalities of the CTI led to prolonged ablation time. RA pouches were usually associated with a prominent Eustachian valve or Eustachian ridge (5, 6) and were previously found to be more prevalent in patients with enlarged LA and reduced left ventricular systolic function (8). However, there are no reports on how to simply identify this particular anatomical variant of pouch in this population without the above-mentioned imaging modalities.

Our results suggest that RA mapping prior to CTI ablation was associated with a significantly reduced ablation time and fluoroscopy exposure, which may facilitate the ablation procedure and hence interventional success rate. In patients with high BMI and heart failure, there was a prolonged time to reach CTI block, and a high number of ablation applications. Those patients were also exposed to increased amount of fluoroscopy. This may be at least partially related to poor catheter stability due to poor respiratory compensation during catheter ablation aside from CTI abnormalities. A higher incidence of AFL has also been reported in patients with chronic obstructive pulmonary disease (COPD) (9). Furthermore, it has been shown that patients with heart failure have a more remodeled CTI, manifesting as prolonged or concave deformation of this region (3). A study using modified long-axis 2D TTE view also reported that the presence of a long CTI, extensive Eustachian ridge, RA pouch and the presence of tricuspid regurgitation were associated with ablation difficulties (10). In our study, lower fluoroscopic exposure and shorter ablation duration were documented in the population with the FRAM protocol, suggesting that FRAM might guide efficient ablation by identifying abnormal CTI anatomy intraoperatively. Although 3D TEE was reported to detect specific pouch structures, this detailed examination is not routine in most clinical centers due to its time-consuming nature (5). ICE is helpful for identification of CTI structures during the intervention (6), but it adds further cost to the procedure. For these reasons, FRAM protocol with CARTO mapping may be considered a convenient approach facilitating the ablation procedure.

Notably, our study revealed that the CHA_2_DS_2_-VASc score showed correlations with increased difficulty of CTI ablation (11). Risk factors present in the CHA_2_DS_2_-VASc score have been associated with atypical AFL including hypertension, cardiomyopathy, prior atrial fibrillation, prior surgery, and COPD (12, 13). Hence, there is an increased likelihood that these patients develop both typical and atypical AFL, either with concurrent atypical AFL or presenting a clinical ECG that is misinterpreted as typical AFL (14, 15). In these cases, a single CTI ablation with bidirectional block was not able to solve the problem. The FRAM protocol in our study not only showed advantages for patients with high CHA_2_DS_2_-VASc score in terms of ablation parameters, but also enabled detection and ablation of concurrent atypical AFL.

This study is a small, single-center, observational cohort study. The correlation coefficient sizes are due to the size of our study population. Our follow-up was rather short and consisted of only ECG/Holter recording and symptom evaluation. The recurrence identification was only based on readmission due to typical AFL. Thus, our follow-up may overestimate the success rate of CTI ablation.

In conclusion, in patients with CTI-dependent AFL, the FRAM approach for CTI ablation, which was originally intended for practical training of our electrophysiology fellows in our center, was associated with reduced ablation duration and fluoroscopy exposures. These advantages may be especially important in patients with high BMI, high CHA_2_DS_2_-VASc scores and reduced left ventricular systolic function.

## Data Availability

The original contributions presented in the study are included in the article, further inquiries can be directed to the corresponding author.
